# G-protein coupled receptor 15 mediates angiogenesis and cytoprotective function of thrombomodulin

**DOI:** 10.1038/s41598-017-00781-w

**Published:** 2017-04-06

**Authors:** Bin Pan, Xiangmin Wang, Chie Nishioka, Goichi Honda, Akihito Yokoyama, Lingyu Zeng, Kailin Xu, Takayuki Ikezoe

**Affiliations:** 1 Department of Hematology and Respiratory Medicine, Kochi Medical School, Kochi University, Nankoku, Kochi Japan; 2 Department of Hematology, Fukushima Medical University, Fukushima, Japan; 3 Department of Hematology, The Affiliated Hospital of Xuzhou Medical University, Xuzhou, Jiangsu China; 4 Medical Affairs Department, Asahi Kasei Pharma., Kanda Jinbocho, Chiyoda-ku, Tokyo Japan

## Abstract

Thrombomodulin (TM) stimulates angiogenesis and protects vascular endothelial cells (ECs) via its fifth epidermal growth factor-like region (TME5); however, the cell surface receptor that mediates the pro-survival signaling activated by TM has remained unknown. We applied pull-down assay followed by MALDI-TOF MS and western blot analysis, and identified G-protein coupled receptor 15 (GPR15) as a binding partner of TME5. TME5 rescued growth inhibition and apoptosis caused by calcineurin inhibitor FK506 in vascular ECs isolated from wild type (WT) C57BL/6 mice. On the other hand, TME5 failed to protect ECs isolated from GPR15 knockout (GPR15 KO) mice from FK506-caused vascular injury. TME5 induced activation of extracellular signal-regulated kinase (ERK) and increased level of anti-apoptotic proteins in a GPR15 dependent manner. In addition, *in vivo* Matrigel plug angiogenesis assay found that TME5 stimulated angiogenesis in mice. TME5 promoted endothelial migration *in vitro*. Furthermore, TME5 increased production of NO in association with activated endothelial NO synthase (eNOS) in ECs. All these pro-angiogenesis functions of TME5 were abolished by knockout of GPR15. Our findings suggest that GPR15 plays an important role in mediating cytoprotective function as well as angiogenesis of TM.

## Introduction

Thrombomodulin (TM), a membrane protein comprising of an N-terminal lectin-like domain (TMD1), a six-tandem epidermal growth factor (EGF)-like region, an O-glycosylation site-rich region, a transmembrane region, and a cytoplasmic tail, is constitutively expressed on vascular endothelial cells and acts as an anticoagulant by binding to thrombin via its fourth to sixth EGF-like regions (TME456)^1, 2^. Simultaneously, the thrombin/TM complex activates protein C and inhibits coagulation in an activated protein C (APC)-dependent way^3^.

Recombinant human soluble thrombomodulin (rTM) comprises the extracellular regions of TM which also counteracts coagulation. A phase III clinical trial proved the safety and efficacy of rTM in patients undergoing disseminated intravascular coagulation (DIC), and showed the superiority of rTM to heparin in treating DIC complicated by infection and haematological malignancies^4^. rTM was approved for treatment of DIC in Japan in 2008. Since then, we have rescued DIC patients complicated by various types of underlying diseases, including sinusoidal obstruction syndrome (SOS) and transplantation-associated microangiopathy (TMA), as well as haematopoietic stem cell transplantation (HSCT) related engraftment syndrome (ES), of which ECs damage are basic pathogenesis^5–7^. Treatment with rTM prolongs overall survival of patients with HSCT related coagulopathy compared with those who were not treated with rTM^8^. Surprisingly, use of rTM counteracted with capillary leakage developed in association with SOS and ES. These observations prompted us to investigate the function of rTM in vascular endothelial cells (ECs) and we found that rTM alleviated apoptosis of ECs caused by calcineurin inhibitors including tacrolimus (FK506) and cyclosporine A (CsA) and inflammatory cytokines. This anti-apoptotic effect was associated with up-regulation of myeloid cell leukemia sequence 1 (Mcl-1) proteins, which was mediated by the activation of extracellular signal-regulated kinase (ERK) signal transduction pathway^9^. Further experiments identified the fifth EGF-like region of TM (TME5) exerts endothelial cytoprotective functions of rTM via APC-independent manner^10^. TM also possesses pro-angiogenic activity via its EGF-like domain^9^. The present study aimed to identify the cell surface expressed protein that interacts with TME5 and mediates endothelial cytoprotective as well as pro-angiogenic function of rTM.

## Results

### TME5 interacts with GPR15

**Figure 1:**
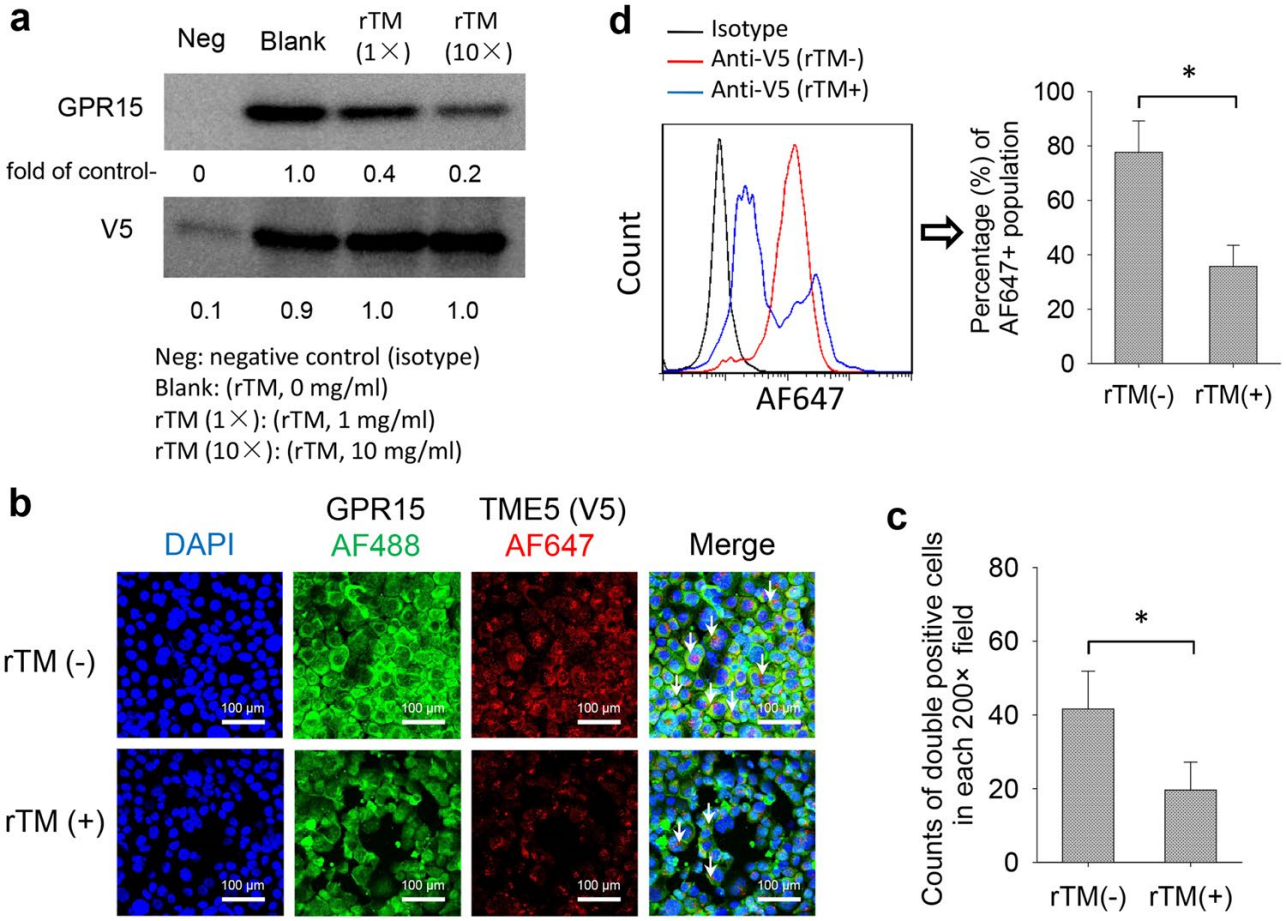
TME5 interacts with GPR15. (**a**) Immunoprecipitation. Membrane proteins isolated from HUVECs were incubated with V5-tagged TME5 (100 μg/ml) in the presence of gradient doses of rTM (0 mg/ml, 1 mg/ml, 10 mg/ml), followed by immunoprecipitation with anti-V5 antibody. Mouse derived isotype antibody was used as negative control. The precipitated proteins were subjected to Western blot analysis to detect the indicated proteins. Figure represents one of the three independent experiments. Relative intensities of bands were analyzed with ImageJ software. (**b–d**) EA.hy926 cells were pre-incubated with either rTM or control diluent followed by incubation with V5-tagged TME5 overnight. (**b**) Immunocytochemistry. Cells were fixed on slide glasses by Autosmear. The fixed cells were stained with anti-GPR15 and V5 antibodies followed by staining with Alexa Fluor 488- and Alexa Fluor 647-conjugated second antibodies. The nuclei were stained with 4′,6-Diamidino-2-phenylindole dihydrochloride (blue). (**c**) Alexa Fluor 488- and Alexa Fluor 647-double positive cells were indicated by white arrows and were counted on 6 random areas under a microscope. (**d**) Flow cytometric analysis. Cells were incubated with anti-GPR15 and V5 antibodies followed by staining with Alexa Fluor 488- and Alexa Fluor 647-conjugated second antibodies. Cell were analyzed on a flow cytometer. Figure shows percentage of Alexa Fluor 647-positive population gated on Alexa Fluor 488-positive cells (n = 6). rTM, Recombinant human soluble thrombomodulin; TME5, the fifth EGF-like region of thrombomodulin.

The immunoprecipitation of mixture of human umbilical vein endothelial cells (HUVECs) membrane proteins and V5-tagged TME5 by anti-V5 antibody followed by MALD-TOF MS analysis identified GPR15 as a candidate binding partner of TME5 (Table 1). Western blot analysis found the presence of GPR15 and TME5 in these precipitated proteins (Fig. 1a). rTM competed the binding of TME5 to GPR15 in a dose dependent manner (Fig. 1a). Immunocytochemistry (Fig. 1b and c) and flow cytometric analysis (Fig. 1d) also suggested the binding of TME5 to GPR15 on cell surface of EA.hy926 cells and pre-incubation of EA.hy926 cells with rTM partially prevented TME5 from binding to GPR15.

**Table 1:** Proteins pulled down by TME5 from MALDI-TOF MS analysis.

Gene symbol	Full Name	Molecular weight	Mascot Score	Significance
GPR15	G protein-coupled receptor 15	41 kD	78	*p* < 0.05
TINAG	tubulointerstitial nephritis antigen	55 kD	43	*p* > 0.05
TRIO	trio Rho guanine nucleotide exchange factor	320 kD	38	*p* > 0.05
IFNL3	interferon lambda-3	22 kD	36	*p* > 0.05

### GPR15 mediates cytoprotective function of TME5

**Figure 2:**
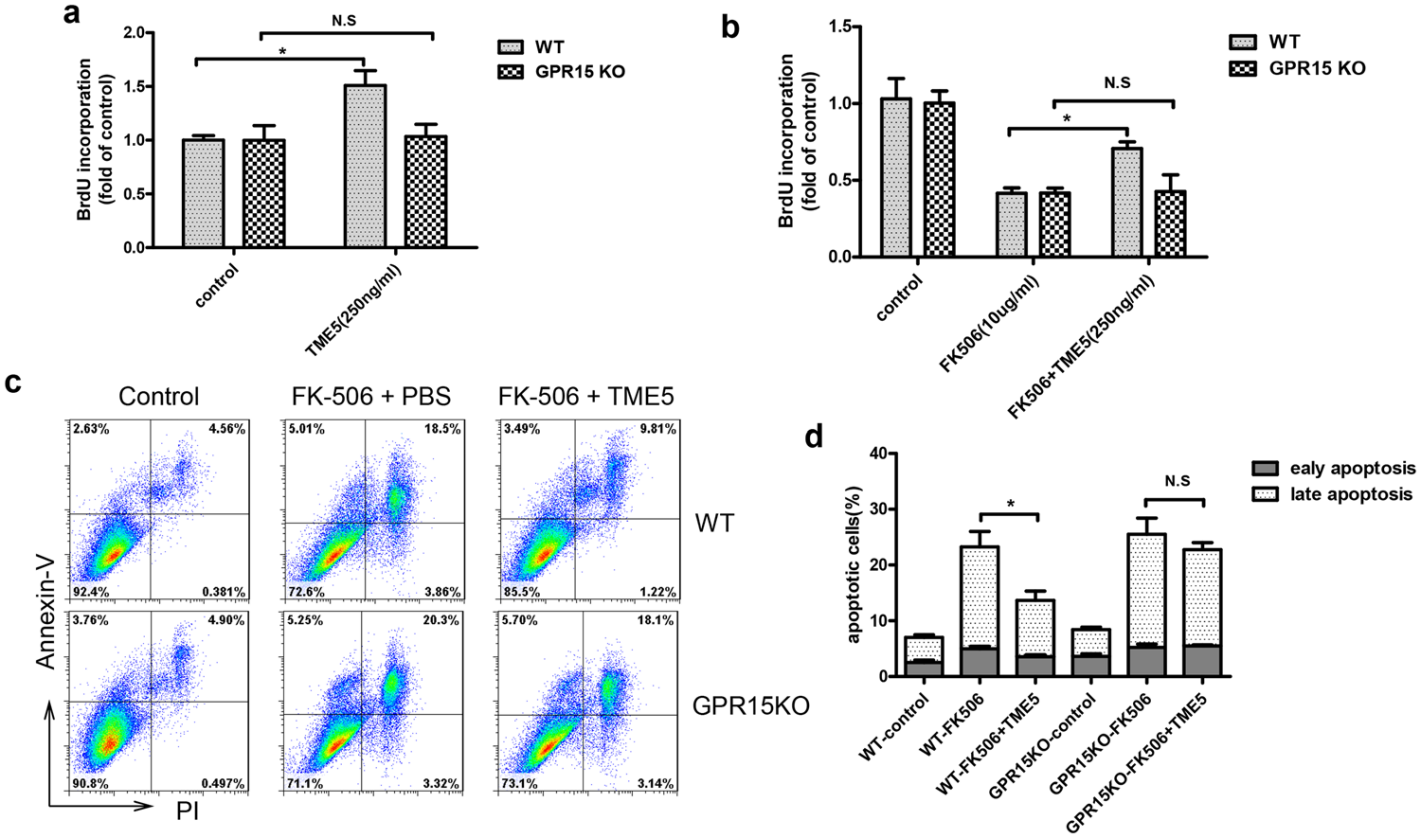
TME5 hampers growth inhibition and apoptosis caused by FK506 in murine vascular ECs in a GPR15 dependent manner. Vascular ECs isolated from wild type (WT) (n = 3) and GPR15 KO (n = 3) mice were cultured with TME5 (250 ng/ml) (**a**) or FK506 (10 μg/ml) or combination of both (**b**) for 24 hrs. Proliferation was measured by bromodeoxyuridine (BrdU) incorporation assays. (**c**) ECs isolated from WT (n = 3) and GPR15 KO (n = 3) mice were exposed to TME5 and/or FK506 (10 μg/ml). After 36 hrs, cells were stained with propidium iodide (PI) and PE-Cy5 anti-annexin V, followed by flow cytometric analysis. Early and late apoptotic cells are indicated by Annexin V + PI− and Annexin V + PI+ cell populations, respectively. Figure represents one from three independent experiments. (**d**) Percentages of apoptotic cells in each group were shown (n = 3). Data are presented as mean ± SD, and compared using one-way ANOVA test. *p < 0.05; N.S., no significance.

Taking advantage of GPR15 KO mice, we next cultured murine aortic ECs (see Supplementary Fig. S1) and performed the functional analysis to test whether GPR15 really mediates the cytoprotective function of TME5. Exposure of vascular endothelial cells to TME5 stimulated the proliferation of ECs isolated from WT C57BL/6 mice by 1.5-fold compared to ECs treated with control diluent (Fig. 2a). On the other hand, TME5 was not able to stimulate the proliferation of ECs isolated from GPR15 KO mice as measured by BrdU incorporation assay (Fig. 2a). FK506, a calcineurin inhibitor, inhibited the proliferation of ECs from both WT and GPR15 KO mice by greater than 50%. Interestingly, FK506-caused inhibition of proliferation of ECs isolated from WT but not from GPR15 KO mice was significantly attenuated in the presence of TME5 (Fig. 2b). Likewise, rTM was able to protect ECs isolated from WT mice from FK506-induced growth inhibition; however, rTM failed to rescue ECs isolated from GPR15 KO mice from insults caused by FK506 (see Supplementary Fig. S2). We also examined whether GPR15 plays a role in TME5-mediated anti-apoptotic effects in ECs. Approximately 25% of ECs isolated from both WT and GPR15 KO mice became apoptotic after exposure to FK506 as assessed by annexin V staining. Intriguingly, TME5 was able to hamper FK506-caused apoptosis in ECs isolated from WT but not from GPR15 KO mice (Fig. 2c and d).

### GPR15 mediates TME5-induced angiogenesis

**Figure 3:**
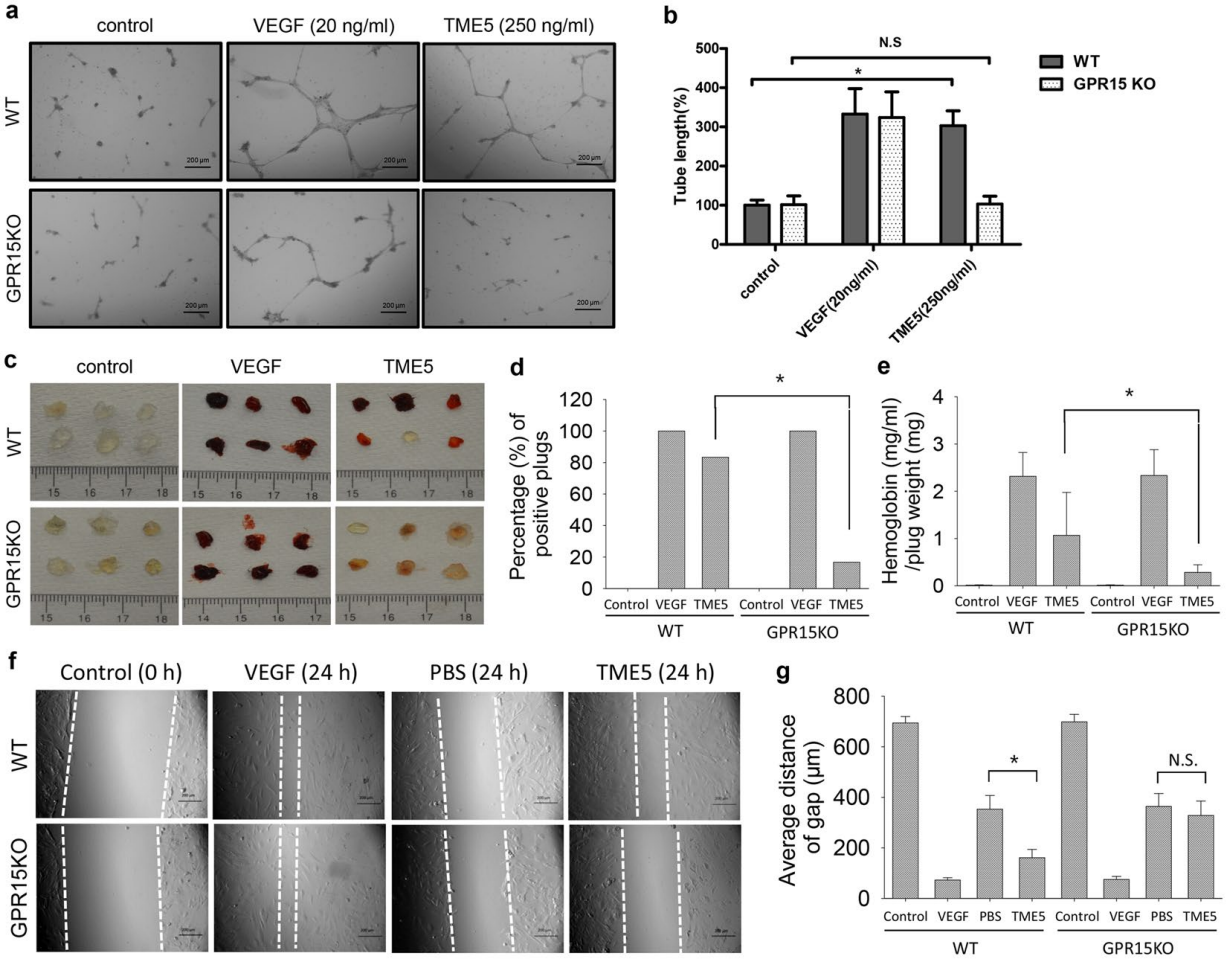
TME5 stimulates angiogenesis of murine ECs in a GPR15-dependent manner. (**a**) *In vitro* vascular tube formation assays. WT (n = 3) or GPR15 KO (n = 3) ECs were plated into a growth factor-reduced Matrigel-precoated 24-well plate and incubated with control diluent, TME5 (250 ng/ml) or VEGF (20 ng/ml). After an 8-hour incubation, the endothelial cell-derived tube-like structure was visualized under an inverted microscope. Figure represents one from three independent experiments. (**b**) The tube length in 3 randomly chosen fields from each well was measured using NIH ImageJ software and normalized to control. (**c**) *In vivo* angiogenesis assays. Growth factor-reduced Matrigel (0.5 ml), containing heparin (40 U/ml), with control diluent, TME5 (250 ng/ml) or VEGF (20 ng/ml) was subcutaneously injected into WT (n = 6) or GPR15 KO (n = 6) mice near the abdominal midline. Five days after injection, mice were euthanized, and the Matrigel plugs were surgically removed. (**d**) Frequencies of positive plugs were expressed as percentage and were compared by using chi-square test. (**e**) Matrigel plugs were weighted and were homogenized in 1 ml distilled water on ice. Supernatants were mixed with Drabkin’s reagent, followed by measurement at 540 nm (n = 6). Methemoglobin was used to create a standard curve. (**f**) Murine ECs were scratched with a 1-ml pipette tip. After being rinsed with warm PBS, plates were supplied with DMEM (5% FBS) medium, containing VEGF (20 ng/ml), PBS or TME5 (250 ng/ml). Plates were photographed at 0 h and 24 h after scratch. (**g**) Average distance of the gaps were calculated from 6 random areas of the scratch. Data are shown as mean ± SD, and are compared using one-way ANOVA test. *p < 0.05; N.S., no significance.

We previously showed that rTM stimulated angiogenesis^10, 11^. Here, we further explored if this proangiogenic effect is preserved in TME5. Vascular endothelial growth factor (VEGF) was used as a positive control to induce angiogenesis. Both VEGF and TME5 increased vascular tube formation in ECs isolated from WT mice (Fig. 3a and b). Similarly, VEGF stimulated vascular tube formation in ECs isolated from GPR15 KO mice. On the other hand, TME5 was not able to induce vascular tube formation in ECs isolated from GPR15 KO mice (Fig. 3a). Similarly, rTM stimulated vascular tube formation of ECs isolated from WT but not from GPR15 KO mice (see Supplementary Fig. S3). Pro-angiogenic effects of TME5 were also examined in Matrigel plug assay in WT and GPR15 KO mice. VEGF stimulated angiogenesis both in WT and GPR15 KO mice in a similar manner (Fig. 3c). On the other hand, TME5 was able to induce angiogenesis only in WT mice (Fig. 3c and d). TME5-treated Matrigel plugs from WT mice contained significantly higher levels of hemoglobin than those from GPR15 KO mice (Fig. 3e).

As endothelial cell migration is a critical step in angiogenesis, we also explored the migration ability of murine ECs. As expected, VEGF induced prompt migration of both WT and GPR15 KO murine ECs. Interestingly, TME5 induced significant migration of WT murine ECs, comparing with PBS treated control. However, TME5 failed to increase migration of GPR15 KO murine ECs (Fig. 3f and g).

### TME5 activates signal transduction pathways via GPR15

**Figure 4:**
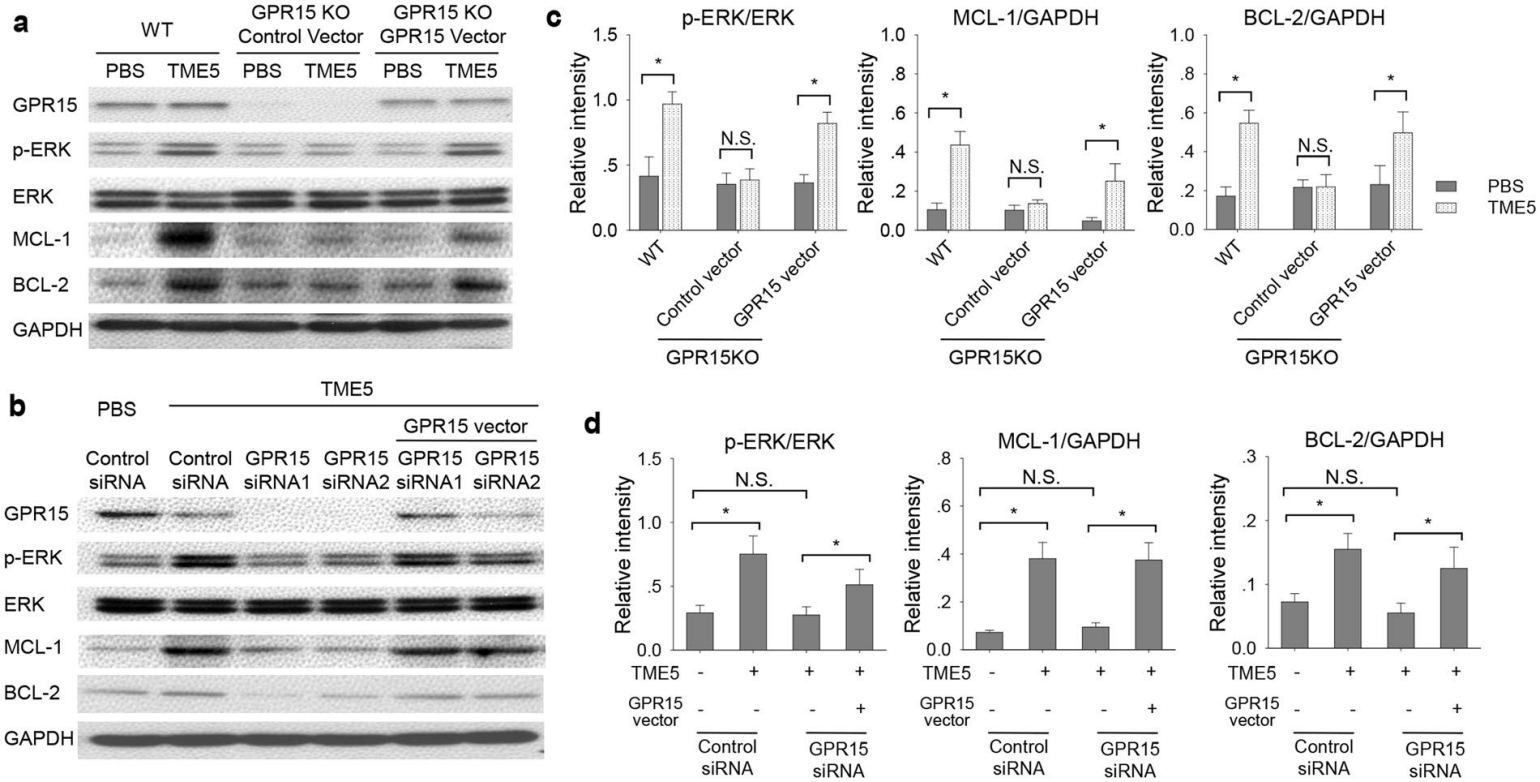
TME5 increases phosphorylation of signal-regulated kinase and level of Bcl-2 in ECs via GPR15. (**a**) Murine ECs from WT or GPR15 KO mice were treated with PBS or TME5 (250 ng/ml) for 48 hrs. In addition, GPR15 KO ECs were transduced with GPR15 expressing lentivirus vector followed by treatment with PBS or TME5 (250 ng/ml). (**b**) HUVECs, transfected with control siRNA or GPR15-specific siRNA, were treated with PBS or TME5 (250 ng/ml) for 48 hrs. For forced expression of GPR15, HUVECs were co-transfected with a GPR15-expression vector. Total proteins extracted from cells were analyzed by western blot. The PVDF membranes were probed with the indicated antibodies in sequence. Figure represents one from three independent experiments. Relative intensities of phospho-ERK, MCL-1 and BCL were analyzed in murine ECs (**c**) and HUVEC (**d**) (n = 3 in each). Data are shown as mean ± SD, and are compared using one-way ANOVA test. *p < 0.05; N.S., no significance.

As rTM increased levels of the phosphorylated forms of ERK and anti-apoptotic protein Mcl-1 in HUVECs^9^, TME5 also increased levels of these proteins in ECs isolated from WT mice (Fig. 4a and c). As expected, TME5 failed to stimulate ERK signaling and increase levels of anti-apoptotic proteins in ECs isolated from GPR15 KO mice (Fig. 4a). Of note, when expression of GPR15 was restored in ECs isolated from GPR15 KO mice by transduction of the GPR15 expression vector, TME5 was able to increase levels of p-ERK in association with increased levels of Mcl-1 and Bcl-2 (Fig. 4a). Similarly, TME5 induced phosphorylation of ERK as well as upregulation of Mcl-1 and BCL-2 in HUVECs, depending on expression of GPR15 on HUVECs (Fig. 4b and d).

### TME5 promotes NO production in a GPR15 dependent manner

**Figure 5:**
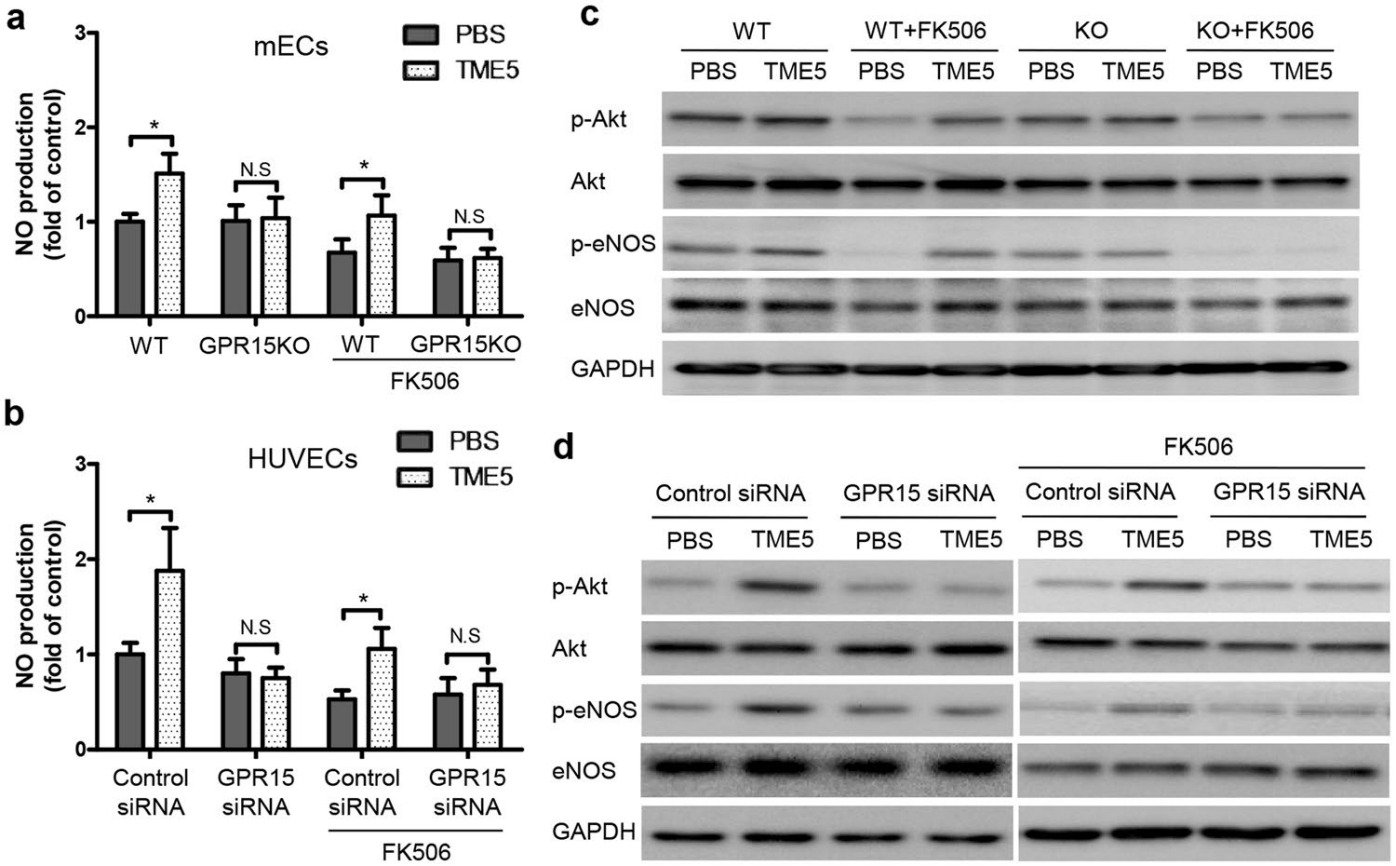
TME5 promotes production of NO in ECs. Murine ECs (**a**) as well as HUVECs (**b**) were incubated with PBS or TME5 (250 ng/ml) for 48 hrs, in the absence or presence of FK506 (10 μg/ml). Supernatants were mixed with Griess reagent followed by reading the absorbance at 540 nm (n = 3 in each). Data are shown as mean ± SD, and are compared using one-way ANOVA test. *p < 0.05; N.S., no significance. Total proteins from murine ECs (**c**) and HUVECs (**d**) were analyzed by western blot with the indicated antibodies. Figure represents one from three independent experiments.

NO plays a crucial role in regulating angiogenesis^12^. FK506 induces ECs dysfunction through suppressing production of NO^13^. TME5 increased NO levels in culture media of both mECs and HUVECs. FK506 reduced NO levels in ECs culture media, which were restored by administration of TME5. TME5 failed to promote production of NO in GPR15-defect ECs (Fig. 5a and b). Endothelial NO synthase (eNOS) is responsible to form NO in ECs^12^. Activation of protein kinase Akt was shown to directly phosphorylate eNOS^14^. We also explored activation of Akt and eNOS in ECs. TME5 increased phosphorylation of Akt and activation (Ser1177) of eNOS either in the absence or in the presence of FK506. However, TME5 failed to induce phosphorylation of Akt and eNOS in GPR15-defect ECs (Fig. 5c and d).

### TME5 suppressed production of pro-inflammatory cytokines in FK506-treated murine ECs

**Figure 6:**
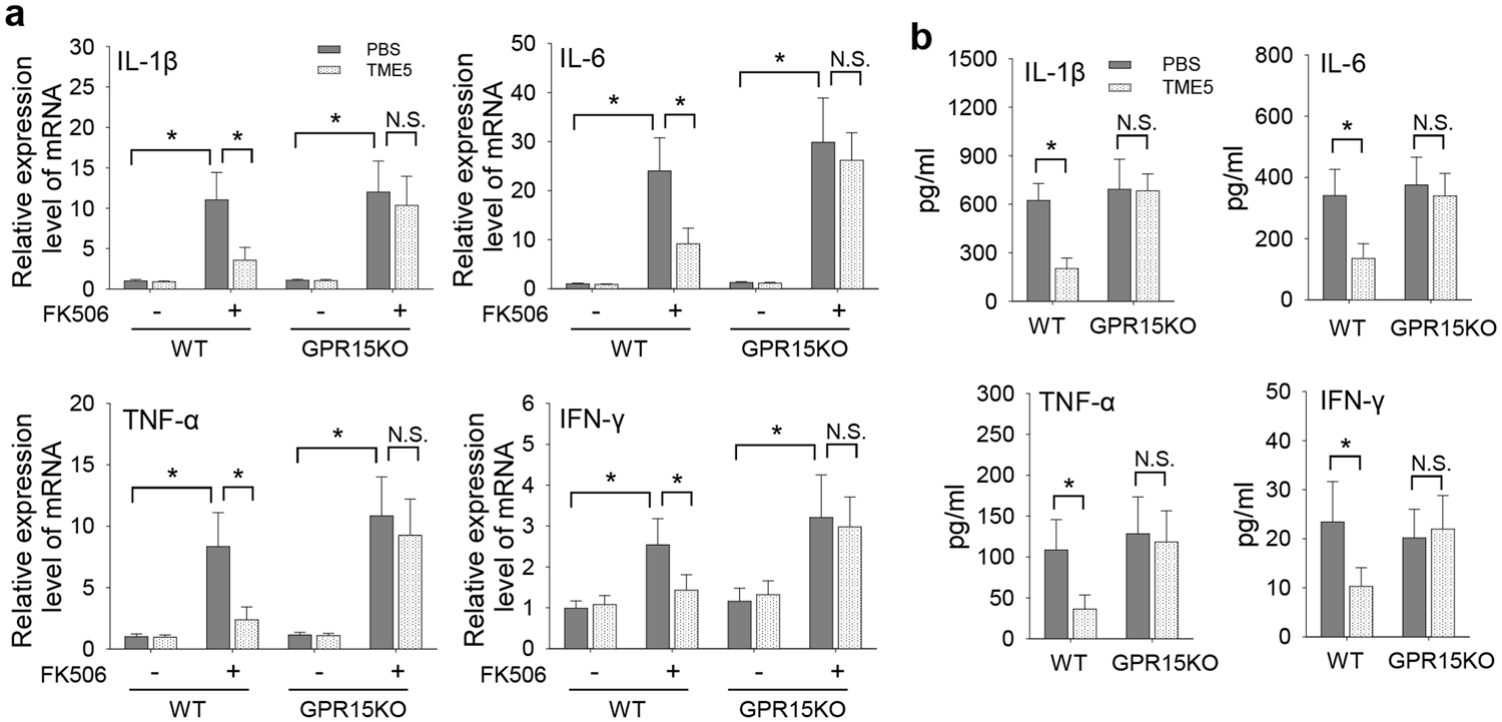
TME5 suppressed production of pro-inflammatory cytokines in FK506-treated murine ECs. Murine ECs from WT or GPR15 KO mice were treated with FK506 (10 μg/ml), in the presence of PBS or TME5 (250 ng/ml) for 48 hrs. (**a**) RNAs were extracted from cells and were subjected to qPCR analysis for mRNA levels of IL-6, IL-1β, TNF-α and IFN-γ. Data represent fold changes (n = 6 in each). (**b**) Supernatants of ECs culture were analyzed by ELISA or cytometric bead array for determining levels of IL-1β, IL-6, TNF-α and IFN-γ. Data are shown as mean ± SD, and are compared using one-way ANOVA test. *p < 0.05; N.S., no significance.

Inflammatory cytokines also contribute to dysfunction of ECs. We previously found that calcineurin inhibitor induced production of pro-inflammatory cytokines in ECs and destructed vascular integrity^15^. We further investigated the impact of TME5 on cytokine production of murine ECs. FK506 induced up-regulation of IL-6, IL-1β, TNF-α and IFN-γ in WT ECs, which were suppressed by using of TME5. However, TME5 failed to inhibit production of these cytokines in GPR15KO ECs (Fig. 6a and b).

## Discussion

The present study employed the proteomic analysis and identified GPR15 as a binding partner of TME5. Other GPR family member such as protease-activated receptor-1 (PAR1) and sphingosine 1-phosphate receptor (S1P_1_) mediate cytoprotective function of APC in vascular ECs^16–18^. TME5 does not produce APC. Thus, TME5-induced angiogenesis and cytoprotective effects are independent of APC.

GPR15 was shown to regulate migration of FOXP3-expressing regulatory T cells to the large intestine and alleviate inflammation^19^. GPR15 is also expressed on T_H_1 and T_H_17 effector T cells and is involved in the pathogenesis of colitis^20^. In addition, GPR15 expressing on CD4-positive T cells acts as a co-receptor for human immunodeficiency viruses^21^. We found that levels of GPR15 in endothelial cells were comparable to those in T cells (see Supplementary Fig. S4), suggesting the crucial roles of GPR15 in maintaining homeostasis or biological function of vascular endothelial cells. Future experiments are required to clarify the function of GPR15 in endothelial cells.

We have recently found that rTM alleviated graft-versus-host disease (GVHD) in a murine HSCT model^22^. The N-terminal lectin-like domain TMD1 also possesses anti-inflammatory function; it inhibits ERK and nuclear transcription factor kappa B which are intimately involved in cytokine production in inflammatory cells^23^. In addition, TMD1 suppresses inflammation through binding and inactivating high-mobility group box 1 protein (HMGB1), which is a potent inflammatory inducer^24^. Calcineurin inhibitors were shown to increase production of pro-inflammatory cytokines in association with activated nuclear factor-κB pathway^15^. Increased levels of pro-inflammatory cytokines contribute to permeability of vascular endothelium. Herein, we found that TME5 suppressed production of pro-inflammatory cytokines in FK506-treated ECs in a GPR15-dependent manner, which indicates a probable anti-inflammatory function of TME5. We are curious to know if rTM mitigates GVHD via TME5 which could affect the function of regulatory T cells via GPR15.

We and others showed that EGF-like domain of TM induced angiogenesis *in vitro* and *in vivo* in association with activation of signal transduction pathways including ERK, AKT and p38 map kinase^9, 11^. Intriguingly, the lectin-like domain TMD1 inhibits ERK signaling and blocks angiogenesis^25^. We previously showed that TME45 stimulated angiogenesis via APC-independent manner^10^. The present study found that pro-angiogenic activity of TM is preserved in TME5 and this activity is also mediated by GPR15, as TME5 was not able to induce angiogenesis in GPR15 KO mice (Fig. 3). NO plays critical roles in regulating angiogenesis. ECs-derived eNOS regulates production of NO either in physiological conditions or under stress^26, 27^. Protein kinase Akt directly activates eNOS and promotes production of NO in ECs^14^. Pleiotropic Akt is involved in transducing signals from G protein coupled receptors (GPCR) family^26^. Our findings suggested that TME5, in the presence of GPR15, induced activation of Akt and eNOS, and thus increased production of NO.

Notably, except for GPR15, a recent *in vitro* study identified fibroblast growth factor receptor (FGFR) as a possible receptor for TM^28^. Our future study will explore if TME5 binds to FGFR.

Taken together, we for the first time demonstrated that TM binds to GPR15 via its EGF-like domain and exerts angiogenesis and cytoprotective function in vascular ECs. The use of ligands interacting with GPR15 may be a promising strategy to prevent or treat lethal complications based on vascular EC damage after haematopoietic stem cell transplantation.

## Methods

### Cells

HUVECs (Lonza, Walkersville, MD) were culture in EGM-2 medium (Lonza). Human endothelial EA.hy926 cells (ATCC, Manassas, VA, USA) were cultured in DMEM medium (Thermo Fisher Scientific, Waltham, MA, USA) containing 10% FBS (Biowest, Nuaillé, France).

### GPR15 knockout mice

*Gpr15* knockout (GPR15 KO) mice (129/SvEv;*129P2-Gpr15* ^*tm1.1Litt*^/*J*, stock number 008769) were purchased from Jackson Laboratory (Bar Harbor, ME). This strain had been backcrossed to C57BL/6 for 3 generations before used for experiments^19^. All procedures regarding animal experiments were approved by the Institutional Animal Care and Use Committee (Kochi Medical School, Kochi University), and all experiments were performed according to its guidelines.

### Reagents

rTM was provided by Asahi Kasei Pharma (Tokyo, Japan). Recombinant mouse VEGF and FK506 were purchased from Peprotech (Rocky Hill, NJ) and Sigma-Aldrich (St. Louis, MO), respectively.

### Generation of TME5

TME5 was produced as previously described^10^. Briefly, TME5 cDNA was amplified by PCR and was cloned into pcDNA3.1/V5-His-A vector (Invitrogen), followed by transfection into COS-1 cells. His-tagged TME5 were purified by using a His-tagged Protein PURIFICATION KIT (MBL, Nagoya, Japan).

### Plasmids and production of proteins

Human GPR15 cDNA was purchased from the Mammalian Gene Collection (BC069437, National Institutes of Health, Bethesda, MD). Murine GPR15 cDNA was purchased from OriGene Technologies (MR217358, Rockville, MD). Both cDNAs were amplified by PCR. Purified products were ligated into the pLenti6.3/V5-TOPO vector (Invitrogen, Carlsbad, CA), followed by transfection into HUVECs. Lentiviral murine GPR15 particles were obtained from 293FT cells, by using a ViraPower™ HiPerform™ Lentiviral Expression System (Invitrogen). Murine ECs were transduced with lentiviral murine GPR15 particles.

### RNA interfering

Three pairs of *Gpr15*-targeting small interfering RNA (siRNA) (SASI_Hs01_00126634, SASI_Hs01_00126636, SASI_Hs01_00126637), synthesized from Sigma-Aldrich (NM_005290, Saint Louis, USA), were transfected into HUVEC with a HUVEC Nucleofector Kit (VPB-1492, Amaxa, Lonza, Walkersville, MD). The efficacy of transfection was approximate 85%. Our preliminary experiment identified SASI_Hs01_00126634 (siRNA-1) and SASI_Hs01_00126636 (siRNA-2) as the most efficient siRNAs in silencing Gpr15 expression, down-regulated by 65% and 70% respectively. A control siRNA (Sigma-Aldrich) was used as negative control.

### Proteomic analysis

Membrane proteins were isolated from HUVECs under non-denaturing conditions using plasma membrane protein extraction kit (BioVision, Milpitas, CA). These proteins were incubated with V5-tagged TME5 overnight followed by immunoprecipitation with anti-V5 antibody (R96025, Life technologies, Carlsbad, CA). The immunoprecipitated proteins were subjected to SDS-PAGE and visualized by SimplyBlue SafeStain (Life technologies). Each band was trypsinized and subjected to MALDI-TOF MS analysis with MALDI-TOF/TOF5800 (AB SCIEX, Tokyo, Japan). These experiments were repeated thrice and identified proteins in at least two experiments were considered as candidate TME5-binding partners. Precipitated proteins were analyzed by western blotting with antibodies to GPR15 (ab8104, Abcam, Cambridge, MA) and V5 (R96025, Life technologies).

### Immunocytochemistry

EA.hy926 cells were fixed on the slide glasses by Autosmear CF120 (Sakura Fine-technical Co. Ltd, Tokyo, Japan). The fixed cells were stained with the first antibodies against GPR15 (ab188938, Abcam, 1:100) and V5 (R96025, Life technologies, 1:100) followed by staining with the secondary antibodies Alexa Fluor 488 (ab150077, Abcam, 1:200) and Alexa Fluor 647 (ab150115, Abcam, 1:200). The nuclei were stained with 4′,6-Diamidino-2-phenylindole dihydrochloride (Roche).

### Culture of murine endothelial cells

Murine endothelial cells (mECs) were isolated from mice as previously described^29^. Briefly, aortic arch and descending aorta were dissected from mice and fat and connective tissues were removed with fine forceps. The dissected aortic vessels were incubated with DMEM (Wako, Tokyo, Japan) in the presence of collagenase type II (Sigma-Aldrich Japan, Tokyo, Japan) for 45 min at 37 degrees Celsius. The cells were harvested and cultured with DMEM supplemented with endothelial cell growth supplement (Sigma-Aldrich, Japan). 5 days later, cells were harvested and utilized for further experiments.

### Proliferation Assay

Proliferation of HUVECs was measured by using BrdU Cell Proliferation kit (Roche, Basel, Switzerland) according to the manual from the manufacturer.

### Nitric oxide production

Nitric oxide (NO) production in ECs culture medium was measured by using Griess reagent (G4410, Sigma-Aldrich) according to manufacturer’s instructions.

### Western blot

Western blot analyses were performed as we described previously^9^. Antibodies to ERK (Cell Signaling Technology, 9102), phospho-ERK (p-ERK) (T202/Y204, Cell Signaling Technology, Danvers, MA, 9101), Mcl-1 (Santa Cruz Biotechnology, Santa Cruz, CA, sc-819), Bcl-2 (Santa Cruz Biotechnology, sc-509), GPR15 (Abcam, ab8104), eNOS (Cell Signaling Technology, 9572), p-eNOS (Ser1177) (Cell Signaling Technology, 9570), AKT (Cell Signaling Technology, 9272), p-AKT (Ser473) (Cell Signaling Technology, 9271) and GAPDH (Cell Signaling Technology, 5174) were used.

### Flow cytometry

Apoptotic cells were quantified using the Annexin V Apoptosis Detection Kit (K129, BioVision, Milpitas, CA) and propidium iodide as previously described^9^.

For analyzing interaction of GPR15 and TME5, EA.hy926 cells were pre-incubated with either rTM or control diluent followed by incubation with V5-tagged TME5 overnight. Cells were incubated with anti-GPR15 and V5 antibodies followed by staining with Alexa Fluor 488- and Alexa Fluor 647-conjugated secondary antibodies.

For analyzing expression of CD31 on murine ECs, cells were trypsinized followed by staining with anti-CD31 (102407, BioLegend, San Diego, CA).

Data were acquired on a BD LSRFortessa flow cytometer and analyzed by using FlowJo software.

### Vascular tube formation assay

To evaluate the effect of TME5 on endothelial tube formation *in vitro*, WT and GPR15 KO mice ECs were plated into growth factor–reduced Matrigel (Corning corporation, New York, NY) precoated 24-well plate at a density of 2 × 10^4^ cells per well and incubated with control diluent, TME5 (250 ng/mL), or vascular endothelial growth factor (20 ng/mL) as a positive control. After 8 hrs, the endothelial cell-derived tube-like structure was visualized under an inverted microscope and photographed at a magnification of ×20. The tube length in 5 randomly chosen fields from each well was measured using NIH ImageJ software and compared with the control.

### Murine angiogenesis assay

To assess the angiogenic effects of TME5 *in vivo*, growth factor–reduced Matrigel (0.5 ml) containing heparin (40 U/mL) with control diluent (double distilled water), TME5 (250 ng/mL), or vascular endothelial growth factor (20 ng/mL) was subcutaneously injected into C57BL6 or GPR15KO mice (6-week-old female) near the abdominal midline. Four days after implantation, mice were euthanized, and the Matrigel plugs were surgically removed and photographed.

### Measurement of hemoglobin

Matrigel plugs were weighted and were homogenized in 1 ml distilled water on ice, followed by centrifugation. Supernatants were mixed with Drabkin’s reagent (Sigma-Aldrich, Japan), and were measured at 540 nm on a plate reader. Methemoglobin was used to create a standard curve^30^.

### Migration assay of endothelial cells

Murine ECs were seeded into 12-well plates to reach a 90% confluence, followed by scratch with a 1-ml pipette tip. After being rinsed with warm PBS, plates were supplied with DMEM medium containing 5% FBS. VEGF, PBS or TME5 were added into different wells, followed by a 24-hour culture. Plates were photographed at 0 h and 24 h. Average distance of the gaps were calculated from 6 random areas of the scratch.

### Quantitative polymerase chain reaction (qPCR)

Total RNA isolation and cDNA synthesis were performed as described previously^10^. Relative expression levels of mRNA were measured by qPCR with a FastStart Universal SYBR Green Master kit (04913914001, Roche). GAPDH was used as a house keeping gene. The results were analyzed by using the method of comparison on −ΔΔct values. The following primers were synthesized in Sigma-Aldrich (Hokkaido, Japan): IL-1β (GGTCAAAGGTTTGGAAGCAG and TGTGAAATGCCACCTTTTGA), TNF-α (AGGGTCTGGGCCATAGAACT and CCACCACGCTCTTCTGTCTAC), IFN-γ (TGAGCTCATTGAATGCTTGG and ACAGCAAGGCGAAAAAGGAT), IL-6 (TGGTACTCCAGAAGACCAGAGG and AACGATGATGCACTTGCAGA) and GAPDH (TTGATGGCAACAATCTCCAC and CGTCCCGTAGACAAAATGGT). qPCR was performed on a StepOnePlus Real-Time PCR System (Thermo Fisher Scientific).

### Measurement of cytokines

Supernatants of ECs culture were collected. Contents of IL-1β were measured by using a mouse IL-1β ELISA kit (R&D system, Minneapolis, MN). Contents of IL-6, TNF-α and IFN-γ were measured by using a cytometric bead array kit (BD Biosciences, San Jose, CA).

### Sorting of T cells

T cells were sorted from spleen of mice by using AutoMACS (Miltenyi Biotec, San Diego, CA). Spleens were grinded with two glasses in PBS. Single-cell suspensions were obtained by passing the suspensions through a Falcon Cell Strainer (352350, Corning, NY). Lymphocytes were isolated by using Lympholyte-M (CL5035, Cedarlane) according to the handbook. T cells were sorted from lymphocytes by using a Pan T Cell Isolation Kit II (Miltenyi Biotec).

### Statistical analyses

Numerical data are presented as mean ± standard deviation (SD). Statistical analyses were performed using unpaired Student *t* test or one-way ANOVA test followed by Bonferroni post-tests. Value of *p* < 0.05 was considered statistically significant.

## Electronic supplementary material

Supplementary Information
