# Supplementary material for: G-protein coupled receptor 15 mediates angiogenesis and cytoprotective function of thrombomodulin
Source: Sci Rep. 2017 Apr 6;7:692. doi: 10.1038/s41598-017-00781-w (PMC5429650; doi:10.1038/s41598-017-00781-w)
Supplement: Supplementary file 1 — Supplementary Information [file 41598_2017_781_MOESM1_ESM.doc]

**Supplementary Information**

**Title**: G-protein coupled receptor 15 mediates angiogenesis and cytoprotective function of thrombomodulin

**Authors’ names and affiliations**

Bin Pan ^1^,^2^,^3^,^#^, Xiangmin Wang ^1,2,3,#^, Chie Nishioka ^1,#^, Goichi Honda ^4^, Akihito Yokoyama ^1^, Lingyu Zeng ^3^, Kailin Xu ^3^, and Takayuki Ikezoe ^1,2,*^

^1^Department of Hematology and Respiratory Medicine, Kochi Medical School, Kochi University, Nankoku, Kochi, Japan

^2^Department of Hematology, Fukushima Medical University, Fukushima, Japan

^3^Department of Hematology, The Affiliated Hospital of Xuzhou Medical University, Xuzhou, Jiangsu, China

^4^Medical Affairs Department, Asahi Kasei Pharma., Kanda Jinbocho, Chiyoda-ku, Tokyo, Japan

^#^These authors contributed equally.

*Correspondence to Takayuki Ikezoe, MD, Department of Hematology, Fukushima Medical University, 1 Hikariga-oka, Fukushima City 960-1295, Japan, Tel.: +81-24-547-1190, Fax: +81-24-548-1821, Email: ikezoet@fmu.ac.jp.

**Figure S1. Morphology and purity of murine ECs.** (**a**). Isolated murine aortic ECs were cultured in DMEM medium, supplied with 20% FBS, 2 mM L-Glutamine and 100 μg/ml endothelial cell growth supplements (ECGS). (**b**). Cells were trypsinized followed by staining with anti-CD31 or isotype antibody. Expression of CD31 were analyzed by flow cytometry. Figure represents one from three independent experiments.

**Figure S2. rTM counteracts FK506-induced growth inhibition in murine ECs in a GPR15-dependent manner.** Vascular ECs isolated from wild type (WT) (n=3) and GPR15 KO (n=3) mice were cultured with rTM (1000 ng/ml) or FK506 (10 μg/ml) or combination of both for 24 hrs. Proliferation was measured by bromodeoxyuridine (BrdU) incorporation assays. Data are shown as mean ± SD, and compared using one-way ANOVA test. **, p* < 0.05; N.S., no significance.

**Figure S3. rTM stimulates angiogenesis of murine ECs in a GPR15-dependent manner.** (**a**). *In vitro* vascular tube formation assays. WT (n=3) or GPR15 KO (n=3) ECs were plated into a growth factor-reduced Matrigel-precoated 24-well plate and incubated with control diluent, rTM (1000 ng/ml) or VEGF (20 ng/ml). After an 8-hour incubation, the endothelial cell-derived tube-like structure was visualized under an inverted microscope. Figure represents one from three independent experiments. (**b**). The tube length in 3 randomly chosen fields from each well was measured using NIH ImageJ software and normalized to control. Data are shown as mean ± SD, and compared using one-way ANOVA test. **, p* < 0.05; N.S., no significance.

**Figure S4. Expression levels of GPR15.** Total proteins from murine ECs (mECs) and sorted murine T cells were extracted and subjected to western blot analysis with the indicated antibodies. Figure represents one from three independent experiments.
